# Transcriptomic signatures in cartilage ageing

**DOI:** 10.1186/ar4278

**Published:** 2013-08-23

**Authors:** Mandy Jayne Peffers, Xuan Liu, Peter David Clegg

**Affiliations:** 1 Comparative Musculoskeletal Biology, Institute of Ageing and Chronic Disease, University of Liverpool, Leahurst, Chester High Road, Neston, Wirral CH64 7TE, UK; 2 Centre for Genomic Research, Institute of Integrative Biology, Biosciences Building, Crown Street, University of Liverpool, Liverpool L69 7ZB, UK

## Abstract

**Introduction:**

Age is an important factor in the development of osteoarthritis. Microarray studies provide insight into cartilage aging but do not reveal the full transcriptomic phenotype of chondrocytes such as small noncoding RNAs, pseudogenes, and microRNAs. RNA-Seq is a powerful technique for the interrogation of large numbers of transcripts including nonprotein coding RNAs. The aim of the study was to characterise molecular mechanisms associated with age-related changes in gene signatures.

**Methods:**

RNA for gene expression analysis using RNA-Seq and real-time PCR analysis was isolated from macroscopically normal cartilage of the metacarpophalangeal joints of eight horses; four young donors (4 years old) and four old donors (>15 years old). RNA sequence libraries were prepared following ribosomal RNA depletion and sequencing was undertaken using the Illumina HiSeq 2000 platform. Differentially expressed genes were defined using Benjamini-Hochberg false discovery rate correction with a generalised linear model likelihood ratio test (*P *< 0.05, expression ratios ± 1.4 log_2 _fold-change). Ingenuity pathway analysis enabled networks, functional analyses and canonical pathways from differentially expressed genes to be determined.

**Results:**

In total, the expression of 396 transcribed elements including mRNAs, small noncoding RNAs, pseudogenes, and a single microRNA was significantly different in old compared with young cartilage (± 1.4 log_2 _fold-change, *P *< 0.05). Of these, 93 were at higher levels in the older cartilage and 303 were at lower levels in the older cartilage. There was an over-representation of genes with reduced expression relating to extracellular matrix, degradative proteases, matrix synthetic enzymes, cytokines and growth factors in cartilage derived from older donors compared with young donors. In addition, there was a reduction in Wnt signalling in ageing cartilage.

**Conclusion:**

There was an age-related dysregulation of matrix, anabolic and catabolic cartilage factors. This study has increased our knowledge of transcriptional networks in cartilage ageing by providing a global view of the transcriptome.

## Introduction

Ageing presents huge challenges for society because whilst the lifespan increases, the quality of life faced by individuals in old age is often poor [1]. The musculoskeletal system in particular is severely affected by the ageing process, with many tissues undergoing changes that lead to loss of function and frailty. Articular cartilage is susceptible to age-related diseases, such as osteoarthritis (OA), although it is not an inevitable result of ageing but rather a consequence of a complex inter-relationship between age and further predisposing factors such as obesity [2], injury [3], genetics [4] and anatomical configuration [5].

A number of studies have interrogated ageing cartilage in order to elucidate the underlying mechanisms that contribute to OA. An age-related reduction in response to insulin-like growth factor in rats resulted in a decline in synthetic activity [6]. Furthermore, using whole mouse joints, Loeser and colleagues demonstrated that there was a reduction in extracellular matrix (ECM) gene expression in older sham-operated mice following surgical destabilisation of the medial meniscus [7]. A characteristic of ageing articular cartilage is the reduction in the number of chondrocytes within the tissue [8,9] and there is evidence of chondrocyte senescence [10]. Chondrocyte senescence is believed to be one cause of a decline in the ability of chondrocytes to respond to growth factors; resulting in the anabolic/catabolic imbalance evident in OA [11]. One of the consequences of cell senescence is an alteration in cell phenotype [12] characterised by increased production of cytokines and growth factors. The increase in ageing chondrocytes expressing this phenotype has been proposed to contribute to cartilage ageing and, given the rise in cytokine production in OA, could directly connect ageing to OA development [13]. Furthermore, there is evidence for the role of oxidative damage in cartilage ageing from reactive oxygen species [14,15], which can result in damage to cartilage DNA [16], whilst a link between reactive oxygen species and development of OA has also been established [17]. Hence, the outcome of ageing on chondrocyte function is an inability to maintain homeostasis when stressed.

There is a need to examine and understand the processes and mechanisms involved specifically in cartilage ageing. Whilst some insights into cartilage ageing have been learnt from transcriptome profiling studies in ageing joints using microarrays [7], these data did not identify a specific chondrocyte phenotype associated with ageing alone. Limitations in coverage and sensitivity mean that a significant part of the chondrocyte ageing transcriptomic phenotype is as yet poorly defined. Advances in high-throughput sequencing methodologies are allowing a new approach to studying transcriptomes: massively parallel sequencing of short reads derived from mRNAs known as RNA-Seq [18]. Compared with microarray technologies, RNA-Seq is demonstrated to enable more accurate quantification of gene expression levels [19]. Furthermore, RNA-Seq is an effective approach for gene expression profiling in ageing tissues with a greater dynamic range and the ability to detect noncoding RNAs [20].

Here we examine the effect of ageing on gene expression in cartilage. Using RNA-Seq analysis of RNA extracted from whole cartilage of young and old equine donors, we elucidate the differential transcriptional signatures associated with ageing and identify some of the molecular mechanisms associated with these changes.

## Methods

### Sample collection and preparation

Samples were collected as a byproduct of the agricultural industry. Specifically, the Animal (Scientific Procedures) Act 1986, Schedule 2, does not define collection from these sources as scientific procedures. Ethical approval was therefore not required for this study. Full-thickness equine cartilage from the entire surface of macroscopically normal metacarpophalangeal joints of eight horses was collected from an abattoir. Horses selected were non-Thoroughbred leisure horses. No exercise history was available for the donors. Macroscopic scoring of the metacarpophalangeal joint was measured using a macroscopic grading system as described previously [21] and samples with no macroscopic perturbations were selected (combined score of zero). Subsequent RNA-Seq experiments were undertaken on normal cartilage from four young horses (4 years old) and four old horses (>15 years old).

### RNA extraction

Cartilage from both articular condyles was removed from the underlying subchondral bone with a scalpel blade under sterile conditions into RNAlater (Sigma-Aldrich, Dorset, UK) according to the manufacturer's instructions. Cartilage was pulverised into a powder with a dismembranator (Mikro-S, Sartorius, Melsungen, Germany) following freezing in liquid nitrogen prior to addition of Tri Reagent (Ambion, Warrington, UK). RNA was extracted using the guanidium-thiocyanate-phenol-chloroform technique, as described previously [22]. Briefly, 20 volumes of Tri Reagent were added to the powdered cartilage tissue and incubated at room temperature for 30 minutes. Following centrifugation at 12,000×*g *for 10 minutes at 4°C, 200 μl chloroform was added to the supernatant, mixed and incubated at room temperature for 10 minutes. The aqueous phase was then precipitated following centrifugation at 12,000×*g *for 10 minutes at 4°C using 70% ethanol. RNA was purified using RNeasy spin columns (Qiagen, Crawley, UK) with on-column DNase treatment (Ambion) to remove residual gDNA according to the manufacturer's instructions. RNA was quantified using a Nanodrop ND-100 spectrophotometer (Labtech, Uckfield, UK) and assessed for purity by ultraviolet absorbance measurements at 260 nm and 280 nm.

### RNA-Seq analysis: cDNA library preparation and sequencing

Eight libraries were prepared representing four animals from two groups, young (*n *= 4) and old (*n *= 4). Total RNA was analysed by the Centre for Genomic Research, University of Liverpool, for RNA-Seq library preparation and sequencing using the Illumina HiSeq 2000 platform (Illumina Inc., San Diego, CA, USA). Total RNA integrity was confirmed using an Agilent 2100 Bioanalyzer (Agilent Technologies, Santa Clara, CA, USA). Ribosomal RNA was depleted from eight total RNA samples using the Ribo-Zero™ rRNA Removal Kit (Human/Mouse/Rat; EpiCentre, Madison, WI, USA) following the manufacturer's instructions. cDNA libraries were prepared with the ScriptSeq v2 RNA-Seq library preparation kit (Epicentre) using 50 ng ribosomal-depleted RNA as the starting material and following the manufacturer's protocols. Briefly, ribosomal RNA-depleted sample was fragmented using an RNA fragmentation solution prior to cDNA synthesis. Fragment size of the final libraries and pooled libraries was confirmed using the Agilent 2100 Bioanalyzer software in the smear analysis function.

Fragmented RNA was reverse transcribed using random-sequence primers containing a tagging sequence at their 5' ends. The 3' tagging was accomplished using the Terminal-Tagging Oligo, which features a random nucleotide sequence at its 3' end, a tagging sequence at its 5' end and a 3'-blocking group on the 3'-terminal nucleotide. Terminal-Tagging Oligo randomly annealed to the cDNA, including to the 3' end of the cDNA. Purification of the di-tagged cDNA was undertaken with AMPure™ XP (Agencourt, Beckmann-Coulter, Beverly, MA, USA). The di-tagged cDNA underwent 15 cycles of amplification using polymerase chain reaction (PCR) primer pairs that annealed to the tagging sequences of the di-tagged cDNA. Excess nucleotides and PCR primers were removed from the library using AMPure™ XP (Agencourt, Beckmann-Coulter). The final pooled library was diluted to 8 pmol before hybridisation. The dilute library (120 μl) was hybridised on each of three HiSeq lanes.

### Data processing

The 100-base-pair paired-end reads obtained by RNA-Seq were compiled using manufacturer-provided pipeline software (CASAVA 1.8.2; Illumina Inc., San Diego, CA, USA). Reads were then aligned onto the equine chromosomes with TOPHAT 1.3.2 (John Hopkins University, Baltimore, MD, USA) using default settings. Only uniquely mapped reads retained with less than two mismatches were used for analysis. Quality control of the reads in each lane was undertaken with FASTQC [23].

The R (version 2.15.1) Bioconductor package edgeR (version 2.13.0) [24] was used to identify differentially expressed genes. edgeR models data as a negative binomial distribution to account for biological and technical variation using a generalisation of the Poisson distribution model. Prior to assessing differential expression, data were normalised across libraries using the trimmed mean of M values normalisation method [25]. Genes were deemed differentially expressed with Benjamini-Hochberg false discovery rate-corrected *P *< 0.05 and fold-change ≥1.4 log_2 _[26] using a generalised linear model likelihood ratio test. This represents a 50% linear fold-change; that is, log_2_1.4 = 0.5 or 50%. Statistical analysis on mapped reads was undertaken with a custom Perl script. All sequence data produced in this study have been submitted to the National Centre for Biotechnology Information GEO under Array Express [GEO:E-MTAB-1386].

### Gene ontology and ingenuity pathway analysis

Owing to the minimal annotation for the equine genome, equine genes were converted to their human Ensembl orthologs prior to bioinformatics analysis. Functional analysis of age-related differentially expressed genes was undertaken to evaluate the differences in gene expression due to age. The functional analysis and clustering tool from the Database for Annotation, Visualisation, and Integrated Discovery (DAVID bioinformatics resources 6.7) was used [27].

Networks, functional analyses, and canonical pathways were generated through the use of ingenuity pathway analysis (IPA; Ingenuity Systems, Redwood City, CA, USA) on the list of differentially expressed genes with value-adjusted *P *< 0.05 and ± 1.4 log_2 _fold regulation. Gene symbols were used as identifiers and the Ingenuity Knowledge Base gene was used as a reference for pathway analysis. For network generation, a dataset containing gene identifiers and corresponding expression values was uploaded into the application. Default settings were used to identify molecules whose expression was significantly differentially regulated. These molecules were overlaid onto a global molecular network contained in the Ingenuity Knowledge Base. Networks of network-eligible molecules were then algorithmically generated based on their connectivity. The functional analysis identified the biological functions and diseases that were most significant to the dataset. A right-tailed Fisher's exact test was used to calculate *P *values. Canonical pathways analysis identified the pathways from the IPA library of canonical pathways that were most significant to the dataset.

### Real-time polymerase chain reaction

Samples of RNA from the same pools used for the RNA-Seq analysis were used for real-time (RT)-PCR. M-MLV reverse transcriptase and random hexamer oligonucleotides were used to synthesise cDNA from 1 μg RNA (both from Promega, Southampton, UK) in a 25 μl reaction. PCR was performed on 1 μl of 10× diluted cDNA, employing a final concentration of 300 nM each primer in 20 μl reaction volumes on an ABI 7700 Sequence Detector using a SYBR Green PCR mastermix (Applied Biosystems, Paisley, Scotland, UK). Exon-spanning primer sequences were used that had been validated in previous publications [28,29] or were designed for this study using Primer-Blast; National Centre for Biotechnology Information BLAST searches were performed for all sequences to confirm gene specificity. Oligonucleotide primers were supplied by Eurogentec (Seraing, Belgium). Steady-state transcript abundance of potential endogenous control genes was measured in the RNAseq data. Assays for four genes - glyceraldehyde-3-phosphate dehydrogenase (GAPDH), TATA box binding protein, beta-actin, and 18 ribosomal RNS - were selected as potential reference genes because their expression was unaltered. Stability of this panel of genes was assessed by applying a gene stability algorithm [30] using genorm^PLUS ^(Biogazelle, Zwijnaarde, Belgium) [31]. GAPDH was selected as the most stable endogenous control gene. Relative expression levels were normalised to GAPDH and calculated using the 2^-ΔCt ^method [32]. Standard curves were generated from fivefold serial dilutions for each assay to confirm that all efficiencies were acceptable; within 5% of GAPDH and *R*^2 ^> 0.98. Primers pairs used in this study are presented in Table 1. RT-PCR analysis data were log_10 _transformed to ensure normal distribution and then analysed using Student's *t *test.

**Table 1:** Gene primer sequences used in RNA-Seq validation

Gene	Accession code	Primer sequence
GAPDH^a^	AF157626	F: GCATCGTGGAGGGACTCA
		R: GCCACATCTTCCCAGAGG
TBP^a^	XM_001502211	F: TGCTGCTGTAATCATGAGGGTAA
		R: TCCCGTGCACACCATTTTC
ACTB^a^	AF035774	F: CCAGCACGATGAAGATCAAG
		R: GTGGACAATGAGGCCAGAAT
18S^a^	AJ311673	F: GGCGTCCCCCAACTTCTTA
		R: GGGCATCACAGACCTGTTATTG
RUNX2	XM_001502519	F: TCCCTGAACTCTGCACCAAG
		R: GCCAGGTAGGAGGGGTAAGA
IL7R	NM_001081942	F: GGCTATGCACAGAATGGAGACT
		R: CAACTGGCTGTAGCACGAGA
SRPX	XM_001489643	F: CTGAGAACAAGGGCG-TTGC
		R: CCGGAGCGTTGAGTTTGC
ACSL5	XM_001915998	F: CCTGGGCTCCTATCTCTTGC
		R: CGGAGATGATCCACTCTGGC
DKK	NM_001267802	F: TAGAACCCTGGGACCTCTGG
		R: GTGTCACTTTGCAAGCCTGG
ADAMTS4^b^	NM_001111299	F: CAGCCTGGCTCCTTCAAAAA
		R: CCGCAGGAATAGTGACCACAT
COL1A1^a^	O46388	F: GACTGGCAACCTCAAGAAGG
		R: CAATATCCAAGGGAGCCACA
COL2A1^a^	NM_001081764	F: TCAAGTCCCTCAACAACCAGAT
		R: GTCAATCCAGTAGTCTCCGCTCTT
COL10A1^a^	XM_001504101	F: TGCCCAGTGGACAGGTTTCT
		R: GTCTTTTCGTTTCTAGTCAGATTTTGAA
MMP1^b^	NM_001081847	F: GGTGAAGGAAGGTCAAGTTCTGAT
		R: AGTCTTCTACTTTGGAAAAGAGCTTCTCT
MMP13^b^	NM_001081804	F: CTGGAGCTGGGCACCTACTG
		R: ATTTGCCTGAGTCATTATGAACAAGAT
IL1β^b^	NM_001082526	F: GAGCCCAATCTTCAACATCTATGG
		R: CAGGCTTGGTAAAAGGACTTGGTAT
TNFα^b^	NM_001081819	F: GCTCCAGACGGTGCTTGTG
		R: GCCGATCACCCCAAAGTG
TGFβ^b^	NM_001081849	F: CCCTGCCCCTACATTTGGA
		R: CGGGTTGTGCTGGTTGTACA

ACSL5, acyl-CoA synthetase long-chain family member 5; ACTB, beta-actin; ADAMTS4, a disintegrin and metalloproteinase with thrombospondin motifs 4; COL1A1, collagen type I, alpha 1; COL2A1, collagen type II, alpha 1; COL10A1, collagen type X, alpha 1; DKK1, dickkopf homolog 1; F, forward; GAPDH, glyceraldehyde-3-phosphate dehydrogenase; IL-1β, interleukin 1 beta; IL7R, interleukin 7 receptor; MMP1, matrix metalloproteinase 1; MMP-13, matrix metalloproteinase 13; R, receptor; RUNX2, Runt-related transcription factor 2; 18S, 18 ribosomal RNS; SRPX, Sushi repeat-containing protein; TBP, TATA box binding protein; TGFβ, transforming growth factor beta; TNFα, tumour necrosis factor alpha;. ^a^Primer pairs previously published in [29]. ^b^Primer pairs previously published in [28].

### Statistical analysis

The analyses were undertaken using the software edgeR [24], S-Plus (Tibco Software Inc., Palo Alto, CA, USA), SPSS (IBM, Portsmouth, Hampshire, UK) and Excel (Microsoft, Redmond, WA, USA).

## Results

### Preliminary analysis of RNA-Seq data

Approximately 116 million to 235 million reads were obtained per sample. Low-quality reads were eliminated, resulting in 7 million to 58 million mapped reads (equal to 6.5 to 35% of the total reads). In total, 3 million to 49 million uniquely mapped read pairs were obtained per sample and aligned to the reference sequence of the equine genome (*Equus caballus*; EquCab2.56.pep [33].

Identical reads mapped to the same genomic position were retained as duplicates because these were potentially real reads. The number of genes per read were normalised to reads per kilobase of exon model per million mappable reads; the values were therefore considered the final expression level for each gene [34]. Using the *E. caballus *database, analysis demonstrated that in total 16,635 genes (from a total of 25,180 genes) were expressed in cartilage, which represented 66% of the equine genome. These data were used for subsequent analysis and are comparable with other recent RNA-Seq studies [35].

### Age-related differential gene expression in cartilage

**Figure 1:**
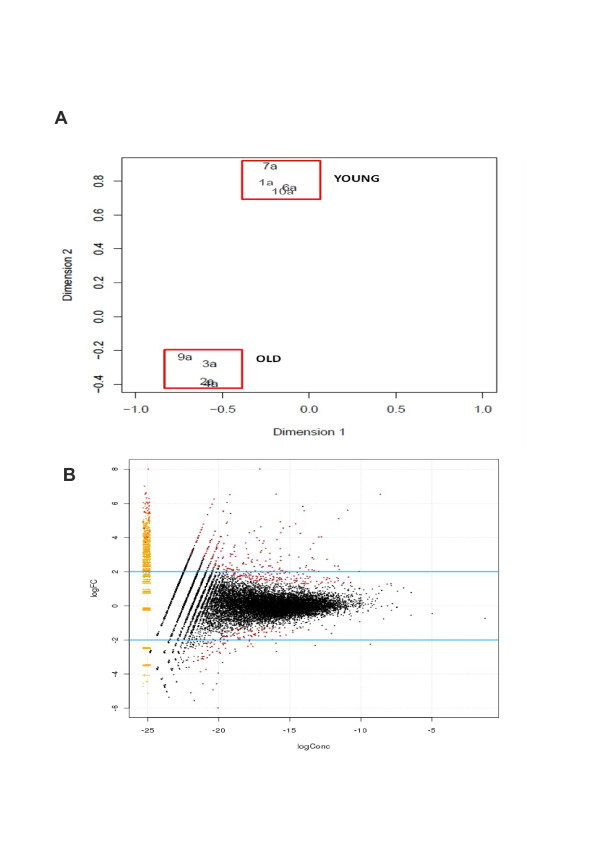
**Principal component analysis and volcano plot of differentially abundant transcripts**. **(A) **Principal component analysis revealed the greatest variability in RNA-Seq data was due to the age of the donor. **(B) **A set of differentially expressed genes between young and old cartilage was discovered. Using the common dispersion in edgeR [24], 396 differentially expressed genes were identified with *P *< 0.05 (red). To enable expression of all genes to be visualised simultaneously, a smear plot was produced. The smear at the left-most edge allows visualisation of genes with zero counts in one of the groups. This was undertaken as if the total counts in one group are zero, the log fold-change is technically infinite, and the log concentration is negative infinity.

A multidimensional scaling plot (Figure 1A) revealed that data were clustered tightly in two groups: one for older donors, and one for younger donors.

Alterations in gene expression between young and old cartilage demonstrated significant age-related changes. There were 396 genes differentially expressed with the criteria *P *< 0.05 and ± 1.4 log_2 _fold-change (Figure 1B); 93 were at higher levels in the older cartilage and 303 were at lower levels in the older cartilage. Table 2 represents the top 10 genes most differentially expressed up and down in the young horses compared with the older horses.

**Table 2:** Genes with the highest and lowest log_2 _fold-change when comparing RNA from young and old cartilage

Gene symbol	Gene name	Log_2 _fold-change	*Q *value
**Genes with increased expression in young cartilage**			
CPZ	Carboxypeptidase Z	32.09	1.60 × 10^-8^
C18orf8	Chromosome 8 open reading frame 4	31.09	1.40 × 10^-4^
SRPX	Sushi repeat-containing protein SRPX	30.69	1.94 × 10^-4^
CYP1A1	Cytochrome P450, family 1, subfamily A, polypeptide 1	30.68	1.19 × 10^-3^
AQP1	Aquaporin 1	30.62	1.19 × 10^-3 ^
PHEX	Phosphate regulating endopeptidase homolog, X-linked	30.39	3.54 × 10^-4^
EPHA5	EPH receptor A5	30.16	1.55 × 10^-^2
CTCFL	CCCTC-binding factor (zinc finger protein)-like	30.15	1.67 × 10^-3^
IL7R	Interleukin 7 receptor	30.14	6.16 × 10^-3^
ACSL5	Acyl-CoA synthetase long-chain family member 5	30.13	2.10 × 10^-2^
**Genes with increased expression in old cartilage**			
SHCBP1L	SHC SH2-domain binding protein 1-like	-3.26	2.66 × 10^-2^
FGF9	Fibroblast growth factor 9	-3.33	4.16 × 10^-4^
SLC22A3	Solute carrier family 22 (extraneuronal monoamine transporter), member 3	-3.73	4.68 × 10^-4^
TOX3	TOX high mobility group box family member 3	-3.86	4.42 × 10^-3^
RELN	Reelin	-4.57	1.87 × 10^-5^
COCH	Coagulation factor C homolog, cochlin (Limulus polyphemus)	-4.57	1.49 × 10^-4^
DKK1	Dickkopf homolog 1	-4.92	6.23 × 10^-4^
LINGO1	Leucine-rich repeat and immunoglobulin domain containing 1	-5.09	2.01 × 10^-2^
SKA1	Spindle and KT associated 1	-5.55	1.55 × 10^-2^
RORA	RAR-related orphan receptor B	-5.98	3.26 × 10^-11^

Log_2 _fold-change and *Q *value (adjusted *P *value) were determined in edgeR [24]. A logarithm to the base 2 of 30 is approximately a linear fold-change of 4.9. Shown are the 10 genes with highest and lowest expression in old compared with young cartilage samples.

**Figure 2:**
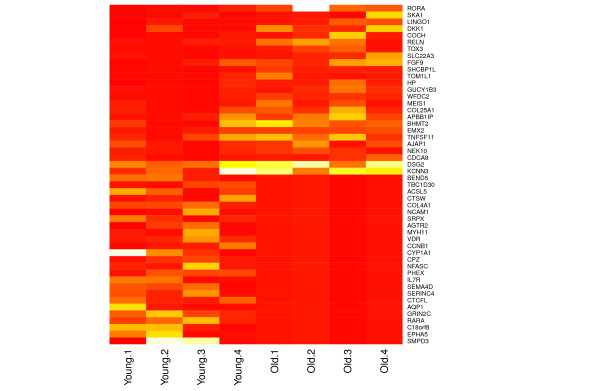
**Top 25 differentially expressed genes in cartilage ageing**. The heat map illustrates the 25 most highly upregulated and downregulated genes in cartilage. The counts represent raw counts for each donor. Significance was set at *P *< 0.05 and ± 1.4 log_2 _fold-change in gene expression based on mapped reads following normalisation and statistical testing in edgeR [24]. Orange, less counts; white, greater number of counts.

The top 25 differentially expressed genes are represented in Figure 2. The National Centre for Biotechnology Information [GEO:E-MTAB-1386] contains a complete list of all genes mapped. The subset of 93 genes that were significantly higher in older donors contained six small nuclear (SNORA)/nucleolar (SNORD) RNAs, 12 pseudogenes, 11 genes that were not identified and a single microRNA (miRNA), miR-21. Thus, 60 known protein coding genes were differentially expressed as higher in the older cartilage. Within the group where gene expression was lower in old compared with young cartilage, nine genes were SNORAs/SNORDs, one was a pseudogene and three were not identified, giving 292 known protein coding genes that were reduced in abundance in older cartilage. Table 3 presents SNORA and SNORDs that displayed age-related differential expression. Thus, 352 genes were used in downstream DAVID and IPA analysis.

**Table 3:** SNORDs and SNORAs identified as being differentially expressed in ageing cartilage.

Name	Family	Action	Target	Log_2 _fold-change	Higher
SNORD113	C/D BOX	Site-specific 2'-O-methylation	Not predicted to target rRNA or snRNA.	29.6	Young
SNORA53	H/ACA box	H/ACA family of pseudouridylation guide snoRNAs	Not identified	29.9	Young
SNORA79	H/ACA box	H/ACA family of pseudouridylation guide snoRNAs	Not identified	5.3	Young
SNORA48	H/ACA box	H/ACA family of pseudouridylation guide snoRNAs	28SrRNA	4.1	Young
SNORD12/SNORD16	C/D BOX	Site-specific 2'-O-methylation	28srRNA, 18SrRNA	3.2	Young
RNase P	RNase MRP related	Site-specific endonuclease, ribosome biogenesis, pre-rRNA processings	Numerous	1.7	Young
Rnase MRP	Rnase MRP	Site-specific endonuclease	Numerous	2	Young
U1 splicesomal RNA	Splicesome	Complex of snRNA and protein subunits that removes introns from a transcribed pre-mRNA		2.3	Young
U2 splicesomal RNA	Splicesome	Complex of snRNA and protein subunits that removes introns from a transcribed pre-mRNA		2.3	Young
SNORA40	H/ACA box class	H/ACA family of pseudouridylation guide snoRNAs	28SrRNA	-1.5	Old
SNORA5	H/ACA box class	H/ACA family of pseudouridylation guide snoRNAs	Not identified	-1.9	Old
SNORA30/SNORA37	H/ACA box class	H/ACA family of pseudouridylation guide snoRNAs	28SrRNA	-2.7	Old
Small nucleolar RNA U89	H/ACA box class	H/ACA family of pseudouridylation guide snoRNAs	Not identified	-1.4	Old
U4 splicesomal RNA	Splicesome	Complex of snRNA and protein subunits that removes introns from a transcribed pre-mRNA		-2.2	Old
U6 splicesomal RNA	Splicesome	Complex of snRNA and protein subunits that removes introns from a transcribed pre-mRNA		-2.1	Old

The class of action and target of these RNAs are shown with higher differential gene expression (DGE) in young or old cartilage. rRNA, ribosomal RNA; snoRNA, small nucleolar RNA.

### Age-related changes in important cartilage genes

There was a reduction in the expression of 42 genes relating to the ECM, degradative proteases, matrix synthetic enzymes, cytokines and growth factors in cartilage derived from older donors compared with young donors. In comparison, there was an increase in only three ECM genes (COL10A1, COL25A1 and lubricin) together with a single growth factor (fibroblast growth factor 9) in older donors (Table 4).

**Table 4:** Older cartilage demonstrated reduced expression of many important cartilage genes compared with young cartilage.

Gene class	Gene name	Gene symbol	Log_2 _fold-change	*Q *value
**DGE higher in old**				
ECM	Collagen, type X, alpha 1	COL10A1	-1.40	3.09 × 10^-2^
	Collagen, type XXV, alpha 1	COL25A1	-2.78	3.62 × 10^-4^
	Lubricin	CSPG4	-2.25	2.53 × 10^-5^
Growth factor/cytokine	Fibroblast growth factor 9	FGF9	-3.33	4.16 × 10^-4^
**DGE lower in old**				
Protease	A disintegrin and metallopeptidase with thrombospondin 12	ADAMTS12	2.20	2.31 × 10^-2^
	A disintegrin and metallopeptidase with thrombospondin 2	ADAMTS2	5.41	1.43 × 10^-11^
	A disintegrin and metallopeptidase with thrombospondin 4	ADAMTS4	1.94	8.47 × 10^-4^
	Matrix metallopeptidase 1	MMP1	1.67	6.00 × 10^-3^
	Matrix metallopeptidase 13	MMP13	2.01	3.07 × 10^-4^
	Plasminogen activator inhibitor-1	SERPINE1	4.24	4.04 × 10^-16^
	Plasminogen activator, tissue	PLAT	1.86	4.19 × 10^-3^
Matrix enzyme	Chondroitin sulfate synthase 3	CHSY3	1.54	2.10 × 10^-2^
	Hyaluronan synthase 3	HAS3	1.64	1.65 × 10^-2^
	Procollagen C-endopeptidase enhancer	PCOLCE	1.90	1.89 × 10^-3^
ECM	Asporin	ASPN	1.55	2.72 × 10^-2^
	Biglycan	BGN	1.47	2.25 × 10^-2^
	Cartilage intermediate layer protein 2	CILP2	4.92	5.97 × 10^-19^
	Chondroadherin	CHAD	2.55	7.89 × 10^-6^
	Collagen alpha 1(V) chain	COL5A1	3.32	9.05 × 10^-11^
	Collagen, type I, alpha 1	COL1A1	6.55	1.49 × 10^-28^
	Collagen, type I, alpha 2	COL1A2	5.57	4.45 × 10^-25^
	Collagen, type II, alpha 1	COL2A1	6.53	1.29 × 10^-32^
	Collagen, type III, alpha 1	COL3A1	5.11	1.04 × 10^-22^
	Collagen, type IV, alpha 1	COL4A1	30.11	6.16 × 10^-3^
	Collagen, type IV, alpha 5	COL4A5	4.07	2.76 × 10^-6^
	Collagen, type IX, alpha 1	COL9A1	8.02	9.70 × 10^-32^
	Collagen, type IX, alpha 2	COL9A2	4.04	2.47 × 10^-15^
	Collagen, type VIII, alpha 1	COL8A1	3.66	3.79 × 10^-10^
	Collagen, type XI, alpha 1	COL11A1	2.01	2.79 × 10^-4^
	Collagen, type XII, alpha 1	COL12A1	1.68	5.11 × 10^-3^
	Collagen, type XIII, alpha 1	COL13A1	4.14	1.23 × 10^-4^
	Collagen, type XIV, alpha 1	COL14A1	3.66	9.07 × 10^-13^
	Collagen, type XV, alpha 1	COL15A1	2.21	7.11 × 10^-5^
	Collagen, type XVI, alpha 1	COL16A1	2.33	1.28 × 10^-5^
	Fibulin 1	FBLN1	4.83	4.44 × 10^-9^
	Fibulin-7	FBLN7	1.66	1.69 × 10^-2^
	Matrilin 2	MATN2	4.56	3.95 × 10^-17^
	Matrilin 4	MATN4	3.69	6.65 × 10^-8^
	Procollagen V, alpha 2	COL5A2	4.03	2.69 × 10^-15^
	Thrombospondin 2	THBS22	2.41	1.26 × 10^-4^
	Thrombospondin 3	THBS3	2.10	1.54 × 10^-4^
Growth factor/cytokine	Fibroblast growth factor 12	FGF12	2.34	1.55 × 10^-2^
	Interleukin-11	IL11	1.48	2.46 × 10^-2^
	Interleukin-8	IL8	1.65	1.41 × 10^-2^
	Interleukin-1b	IL1B	6.26	8.91 × 10^-10^
	Tumour necrosis factor, alpha-induced protein 3	TNFAIP3	1.73	1.49 × 10^-2^

The table illustrates significant differential gene expression (DGE) in young and old cartilage of important cartilage, extracellular matrix (ECM), cytokines and growth factors, proteases (causing cartilage degradation) and matrix enzymes (involved in matrix synthesis). Significance was set at *P *< 0.05 and ± 1.4 log_2 _fold-change in gene expression based on mapped reads following normalisation and statistical testing in edgeR [24].

### Gene ontology analysis of differentially expressed genes to characterise transcriptomic signatures in cartilage ageing

DAVID analysis of all differentially expressed genes included annotations for cell adhesion and the ECM (see Additional file 1). The genes most differentially expressed, with reduced expression in cartilage from older donors, included two involved in Wnt signalling: carboxypeptidase Z and chromosome 8 open reading frame 4. Furthermore, the abundance of three other genes involved in Wnt signalling (secreted frizzled-related protein 2, Wnt11 and Wnt inhibitory factor-1) were also reduced in old cartilage. Interestingly, of the genes expressed in higher levels in older cartilage, one of the most highly regulated was the negative regulator of Wnt signalling, dickkopf homolog 1 (DKK1). DAVID analysis of this group revealed annotations for skeletal and cartilage development, and immune response.

### Differential expressed genes and network analysis

**Figure 3:**
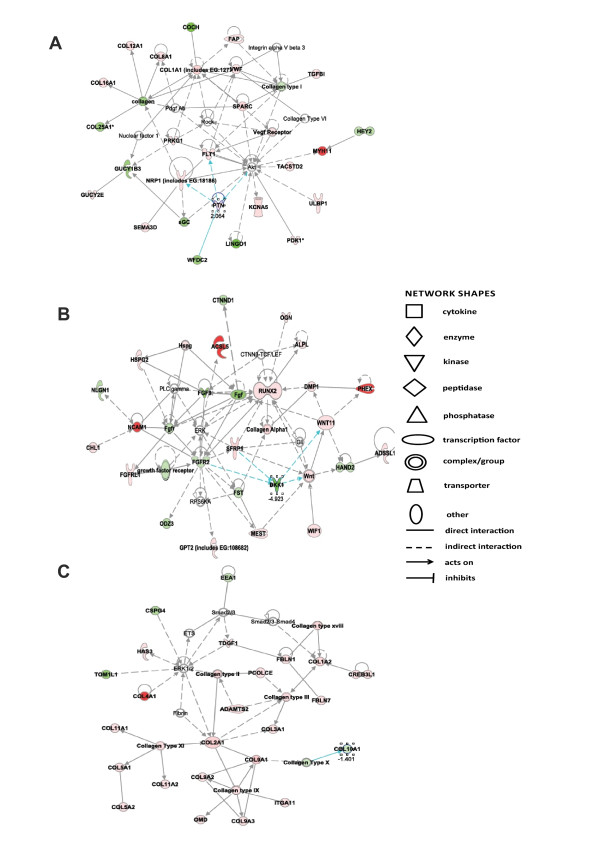
**Top-scoring networks derived from the 352 genes differentially expressed in ageing**. **(A) **Ingenuity pathway analysis (IPA) identified connective tissue disorders as the principle associated network functions with scores of 43. **(B) **The second top-scoring network was a further connective tissue disorder with scores of 35. **(C) **IPA identified ageing significantly affected the connective tissue development and function network in ageing cartilage. Figures are graphical representations between molecules identified in our data in their respective networks. Green nodes, upregulated gene expression in older cartilage; red nodes, downregulated gene expression in older cartilage. Intensity of colour is related to higher fold-change. Key to the main features in the networks is shown.

Both sets of differentially expressed genes associated with ageing were analysed together in IPA with the following criteria; *P *< 0.05 and ± 1.4 log_2 _fold-change. Network-eligible molecules were overlaid onto molecular networks based on information from the ingenuity pathway knowledge database. Networks were then generated based on connectivity. (Additional file 2 contains all identified networks and their respective molecules.) Interesting age-related features were determined from the gene networks inferred. According to the top-scoring network, the differentially expressed genes were from connective tissue disorders, such as collagens COL12A1, COL16A1, COL1A1, and COL25A1 plus leucine-rich repeat and immunoglobulin domain containing 1 (LINGO), transforming growth factor beta (TGFβ)-induced 68 kDa and coclin (COCH) (Figure 3A).

Other networks significantly enriched also related to a further network in connective tissue disorders that contained genes including collagens COL10A1, COL11A1 and COL2A1 plus a disintegrin and metalloproteinase with thrombospondin motifs-2 (ADAMTS-2) and fibulin-1 (FBLN1) (Figure 3B). Additionally, a connective tissue development network was also significantly affected. The genes most affected in this network included acyl-synthetase long chain family member 5 (ACSL5), phosphate-regulating neutral endopeptidase (PHEX) and DKK1 (Figure 3C).

Significant IPA canonical pathways are demonstrated in Table 5 and the associated molecules of the top canonical pathways identified are in Additional file 3. These include atherosclerosis signalling, prothrombin activation and rheumatoid arthritis.

**Table 5:** IPA canonical pathways were significantly affected in ageing cartilage

Name of canonical pathway	*P *value	Ratio
Atherosclerosis signalling	3.80 × 10^-9^	15/136 (0.11)
Role of osteoblasts, osteoclasts and chondrocytes in rheumatoid arthritis	3.41 × 10^-6^	16/238 (0.067)
Intrinsic prothrombin activation	9.82 × 10^-6^	6/35 (0.171)
Hepatic fibrosis and stellate cell activation	9.92 × 10^-6^	12/146 (0.082)
Role of macrophages, fibroblasts and endothelial cells in rheumatoid arthritis	1.73 × 10^-4^	16/333 (0.048)

The significance of the association between the dataset and the canonical pathway was measured using a ratio of the number of molecules from the dataset that mapped to the pathway divided by the total number of molecules that map to the canonical pathway is displayed. Fisher's exact test was used to calculate *P *values.

### Confirmation of differential gene expression using real-time PCR measurements of selected genes

To validate the RNA-Seq technology, 14 genes were selected to measure using reverse transcription and RT-PCR based on differences noted in the arrays and/or their potential importance in the OA process. This was performed on the original RNA from all donors used to perform the RNA-Seq experiment (Table 6). Genes were selected based on differences noted in the RNA-Seq results. All genes were found to have comparable results with RNA-Seq data; for instance, genes identified as having an increase in expression in older samples in the RNA-Seq experiment also gave increased expression relative to GAPDH following RT-PCR. Statistical significance was tested using Student's *t *test. Two genes whose expressions were not significantly altered in RNA-Seq results - tumour necrosis factor alpha and transforming growth factor β (TGFβ) - were also unaltered when assessed with RT-PCR.

**Table 6:** Real-time polymerase chain reaction analysis of 14 selected genes reveals good correlation with RNA-Seq results

Gene	RNA-Seq results			RT-PCR results					
	
	Differential expression	Significant log_2 _fold-change	*Q *value	Age		2^-ΔCt ^log_2 _fold-change	*P *value	Mean Ct value, young	Mean Ct value, old
								
				Young	Old				
DKK1	Higher in old	-4.9	0.0006	0.0044 ± 0.006	0.0338 ± 0.20	-3.0	0.024	30.9	28.8
COL10	Higher in old	-1.4	0.03	0.0006 ± 0.0001	0.0013 ± 0.0009	-1.0	0.26	32.4	32.3
RUNX2	Lower in old	2.2	0.038	0.0093 ± 0.009	0.0034 ± 0.002	1.5	0.002	29	30.8
SRPX	Lower in old	1.6	0.009	0.0046 ± 0.005	0.0006 ± 0.0005	3.0	0.007	30	33.8
ACSL5	Lower in old	30.1	0.02	0.0155 ± 0.0002	0.0062 ± 0.005	1.3	0.09	29.5	29.6
IL7R	Lower in old	30.1	0.06	0.0005 ± 0.0002	0.0001 ± 0.0001	2.3	0.06	33.9	37
COL2A1	Lower in old	6.5	2.29 × 10^-32^	63.3246 ± 54.7	1.3165 ± 1.12	5.7	0.04	18.3	22.4
COL1A1	Lower in old	6.5	1.48 × 10^-28^	3.4815 ± 1.57	0.0278 ± 0.02	7.0	0.15	24.3	27.5
MMP1	Lower in old	1.6	0.006	0.7093 ± 0.21	0.4027 ± 0.14	0.8	0.05	23.1	24
MMP13	Lower in old	2	0.0003	0.2323 ± 0.15	0.044 ± 0.04	2.4	0.1	26.1	26.8
ADAMTS-4	Lower in old	1.9	0.0008	0.5121 ± 0.35	0.1345 ± 0.05	1.9	0.07	24	24.9
IL1β	Lower in old	6.2	8.91 × 10^-10^	0.0057 ± 0.005	0.0004 ± 0.0003	3.9	0.05	30.1	33.4
TNFα	No change	Not significant	1	0.0041 ± 0.004	0.001 ± 0.0003	2.1	0.28	31.1	32.1
TGFβ	No change	Not significant	1	1.2865 ± 0.23	2.3124 ± 1.24	-0.8	0.15	21.9	21.4

Values for real-time polymerase chain reaction (RT-PCR) are the mean ± standard deviation of relative expression levels normalised to expression of glyceraldehyde-3-phosphate dehydrogenase. Statistical significance was tested using Student's *t *test. Log_2 _fold-change of 2^-ΔCt ^values is shown for comparison. Average threshold cycle (Ct) values for young and old donors are demonstrated to indicate robustness of expression. DKK1, dickkopf homolog 1; COL10, collagen type X; RUNX2, Runt-related transcription factor 2; SRPX, Sushi repeat-containing protein; ACSL5, acyl-CoA synthetase long-chain family member 5; IL7R, interleukin 7 receptor; COL2A1, collagen type II, alpha 1; COL1A1, collagen type I, alpha 1; MMP1, matrix metalloproteinase 1; MMP-13, matrix metalloproteinase 13; ADAMTS4, a disintegrin and metalloproteinase with thrombospondin motifs 4; IL-1β, interleukin 1 beta; TNFα, tumour necrosis factor alpha; TGFβ, transforming growth factor beta.

In addition, quantitative RT-PCR was undertaken for the 14 genes on a different set of donors to those used in the RNASeq study in order to validate our findings young (4 years old, *n *= 4) and old (>15 years old, *n *= 4) (Table 7). All genes were found to have comparable results.

**Table 7:** Real-time PCR analysis of 14 selected genes using a different set of donors reveals similar correlation with RNA-Seq results

Gene name	Differential expression	Age		*P *value
			
		Young	Old	
DKK1	Higher in old	0.0004 ± 0.0006	0.008 ± 0.0006	0.04
COL10	Higher in old	7.66 × 10^-5 ^± 3.05 × 10^-5^	0.000148 ± 7.13 × 10^-5^	0.1
RUNX2	Lower in old	0.002 ± 0.0004	0.0005 ± 0.0004	0.02
SRPX	Lower in old	0.0025 ± 0.0002	0.001 ± 0.002	0.05
ACSL5	Lower in old	0.004 ± 0.002	0.002 ± 0.02	0.13
IL7R	Lower in old	0.001 ± 0.0002	0.0007 ± 0.0004	0.07
COL2A1	Lower in old	32.6 ± 24.5	0.2 ± 0.13	0.04
COL1A1	Lower in old	0.71 ± 0.11	0.02 ± 0.02	0.0003
MMP1	Lower in old	0.28 ± 0.17	0.07 ± 0.03	0.03
MMP13	Lower in old	0.08 ± 0.12	0.02 ± 0.02	0.28
ADAMTS-4	Lower in old	0.07 ± 0.05	0.03 ± 0.02	0.07
IL1β	Lower in old	0.0001 ± 0.0002	2.66 × 10^-5 ^± 1.14 × 10^-5^	0.03
TNFα	No change	0.0001 ± 7.7 × 10^-5^	9.8 × 10^-5 ^± 4.5 × 10^-5^	0.4
TBFβ	No change	0.58 ± 0.14	1.06 ± 0.5	0.12

Values for real-time polymerase chain reaction (RT-PCR) are the mean ± standard deviation of relative expression levels normalised to expression of glyceraldehyde-3-phosphate dehydrogenase. Statistical significance was tested using Student's *t *test. DKK1, dickkopf homolog 1; COL10, collagen type X; RUNX2, Runt-related transcription factor 2; SRPX, Sushi repeat-containing protein; ACSL5, acyl-CoA synthetase long-chain family member 5; IL7R, interleukin 7 receptor; COL2A1, collagen type II, alpha 1; COL1A1, collagen type I, alpha 1; MMP1, matrix metalloproteinase 1; MMP-13, matrix metalloproteinase 13; ADAMTS4, a disintegrin and metalloproteinase with thrombospondin motifs 4; IL-1β, interleukin 1 beta; TNFα, tumour necrosis factor alpha; TGFβ, transforming growth factor beta.

## Discussion

Ageing has an important role in the development of OA by making the joint more susceptible to OA risk factors. To provide interventions to prevent age-related changes and reduce the risk of developing OA, the underlying mechanisms involved in age-related changes of cartilage require elucidation. Characterisation of both young and old cartilage at the molecular level is essential for identifying the important signalling pathways in OA development. In the present study, we used the RNA-Seq technique to undertake deep transcriptome profiling of young and old cartilage. This is the first time that, to our knowledge, this technique has been used to interrogate transcriptional changes in cartilage ageing and, importantly, validation studies using RT-PCR demonstrated high correlation between methodologies and demonstrated reproducibility using a different donor set.

This study built on previous findings that identified a reduction in matrix gene expression with joint ageing [7]. We took a single tissue, articular cartilage, and undertook RNA-Seq in order to interrogate a greater range of genes for differential expression. Not surprisingly, our experiments identified that the age of the donor accounted for the principal variability in the data. The major findings of this study were as follows: the age-related gene expression changes identified were most notably involving reduced differential gene expression in older cartilage; there was an over-representation of genes with reduced expression relating to the ECM, degradative proteases, matrix synthetic enzymes, cytokines and growth factors in cartilage derived from older donors compared with young donors; cartilage ageing caused a decrease in many important Wnt signalling genes; IPA revealed that the top-scoring network for differentially expressed genes was from connective tissue disorders and connective tissue development; IPA also demonstrated significant canonical pathways for atherosclerosis signalling, prothrombin activation and rheumatoid arthritis; and there was differential expression of pseudogenes and small noncoding RNAs in cartilage ageing with increased expression of 12 pseudogenes and six noncoding RNAs in older cartilage, and of one pseudogene and nine small noncoding RNAs in younger cartilage.

Equine tissue was readily obtained, enabling collection of cartilage samples from macroscopically normal, skeletally mature young and aged horses. Importantly, the horse suffers clinical joint diseases similar to man (reviewed [36]), and as such has been used as a model for naturally occurring OA [37] due to extensive knowledge of its pathogenesis and clinical experience of the disease [38]. Indeed, the incidence of equine metacarpophalangeal OA in young racehorses [39] in training is similar to the incidence of post-traumatic OA in man [40]. Additionally, the articular cartilage thickness is also comparable between species [41].

For young horses one year is equivalent to about 3.5 years of a human [42,43]. The rate of equine ageing relative to equivalent human age is greatest within the first two years of life and decreases after the horse reaches maturity at 4 years of age [44]. Hence, horses >15 years old, as used in this study, are likely to equate to humans older than 52 years. The average lifespan of a horse is 25 to 30 years and so it is possible that the obvious differences in lifespan may yield significant differences in the effect of ageing amongst animal species due to cumulative lifetime load. However, whilst the work in this study may not be directly applied to humans, it does enable an insight into human cartilage ageing by studying a population at skeletal maturity to one beyond the middle age equivalent in man.

This study utilised the entire articular surface of distal metacarpal III bone. High and low load-bearing cartilage was thus used. An assessment of macroscopic changes revealed no abnormalities in our samples. Previous studies indicated a high correlation between gross scoring and Mankin's grading in equine cartilage from the distal metacarpal III bone [28,45]. To validate that the RNA extracted from the harvested tissue was articular cartilage, the expression level of several genes typically expressed and those of bone were measured. There was a high expression of articular cartilage genes only (data not shown).

Previous studies have identified a number of age-related changes in chondrocyte metabolism (reviewed in [46]). Most of these studies demonstrate changes at the protein level, such as an age-related decline in matrix production when equine chondrocytes were stimulated with TGFβ1 [47]. Others have provided evidence for a chondrocyte senescence secretory phenotype in ageing, demonstrated by an increase in cytokines [48,49] along with matrix metalloproteinase (MMP) production and a reduction in growth factors [50,51]. These studies did not interrogate transcript changes and of course simple deduction of protein from mRNA expression is insufficient because post-translational regulation, small noncoding RNAs, decay differences in mRNA and proteins, and locations or molecular associations of proteins affect overall protein levels [52]. However, a recent whole mouse-joint study demonstrated a reduction in matrix genes with age [7] in agreement with our findings. Furthermore, a study of equine articular cartilage concluded that although there was no change in the age-related expression of MMP-13 there was a reduction in MMP-3 and interleukin (IL)-1β gene expression in cartilage from older donors [53]. Annotations of genes at reduced levels in older samples included many relating to the ECM, degradative proteases, matrix synthetic enzymes, cytokines and growth factors. In contrast, within these annotations those at higher levels in older cartilage were very small: COLX, COLXXV, lubricin and fibroblast growth factor 9.

There appears to be an age-related failure of matrix, anabolic and catabolic cartilage factors. This is interesting because a recent study on postnatal and skeletally mature equine cartilage identified a reduction in collagens, matrix modelling and noncollagenous matrix transcripts with age [54]. ADAMTS-4 expression was reduced in the older cartilage in this study, which is in agreement with findings in ageing rat cartilage [55]. In contrast, previous studies have demonstrated an increase in IL-7 in ageing chondrocytes and in response to fibronectin fragments or IL-1 [49]. Although our experiment did not identify IL-7, interestingly one of the most downregulated genes identified in this study was the IL-7 receptor. A reduction in IL-7 receptor signalling in ageing β-progenitor cells has been demonstrated previously to result in ageing-like gene expression profiles [56]. Also, whereas other studies have demonstrated an increase in IL-1 [48] (where an increase in IL-1 protein was evident in older cultured human chondrocytes) and MMP-13 [48,57] in ageing human cartilage, this study identified an age-related decline in their transcript abundance. However, one MMP-13 study looked at catabolic responsiveness with age whilst another used immunolocalisation of MMP-13 to identify protein. These two factors are not always related [58]. Whilst differences could also be attributed to our age classification of young and old and species distinctions, increased matrix enzymes (MMP-1, MMP-13) and cytokines such as IL-1, IL-8 and IL-11 identified in younger cartilage could be due to increased turnover. Interestingly a recent study identified that low innate capacity to produce IL-1β and IL-6 was associated with the absence of OA in old age [59]. The reduction in IL-1β evident in older cartilage may represent a protective mechanism against OA.

We noted in cartilage derived from old donors that there was primarily a reduction in the expression of some key Wnt signalling genes plus an increase in the Wnt antagonist DKK1 and a reduction in RUNX2, a downstream target of Wnt. Wnt signalling is active in adult cartilage, with deregulation being detrimental, resulting in age-associated joint pathologies due to excessive remodelling and degradation [60]. This signalling pathway has also been found to both regulate matrix synthesis in chondrocyte cell lines [61] and stimulate catabolic genes such as MMP-13 and ADAMTS-4 in chondrocytes [62]. A recent study demonstrated a potential protective function of Wnt in ageing. The activation of the Wnt pathway inhibited IL-1-mediated MMP-13 expression in human chondrocytes through the direct interaction between nuclear factor-κB and β-catenin [63]. One study has linked Wnt signalling with chondrocyte hypertrophy through RUNX2 activation [64], whilst elsewhere it was shown that DKK1 is a major player in the cessation of hypertrophic differentiation that can contribute to OA [65]. Interestingly, COL10A1, a marker of chondrocyte hypertrophy, was increased in old cartilage. However, COL10A1 has also been identified in the transitional zone of cartilage and may have a role in the modification of collagen fibril arrangement [66]. A recent study in mesenchymal stems cells derived from OA patients found that COL10A1 downregulation played a role in the establishment of a defective cartilage matrix in OA [67]. It would seem that this increased expression with ageing is not through the Wnt signalling interaction with subsequent RunX2 activation as described previously [64]. Further credence is given to this hypothesis by our findings that alkaline phosphatase expression, also regulated through RunX2, was downregulated in old cartilage. Overall Wnt signalling is involved in maintenance of cartilage, and the dysregulation event here in ageing may be an important episode. Interfering with the pathway may contribute to improvements in cartilage regeneration.

Using IPA, this study identified age-related changes in pathways and processes including connective tissue disorders and development in which a significant number of genes, regulated both strongly and subtly, were enriched. This is not remarkable given the number of matrix genes differentially identified in the study. Care should also be taken in overinterpretation of this finding because some of the genes in this network are minor components of cartilage, such as COL12A, COL16A, COL25A, LINGO and COCH. Canonical pathways identified as significantly affected by ageing, such as the role of osteoblasts and osteoclasts in rheumatoid arthritis, were not surprising. Interestingly, age-affected atherosclerosis signalling pathways follow the differential expression of a mixture of proteases and lipoproteins. In ageing cartilage, further studies to investigate the significance of this are clearly required.

One advantage for the use of RNA-Seq to undertake differential gene expression studies is that other sets of RNA molecules from the transcriptome can be identified, such as nonprotein coding RNAs (for example, miRNA and small nucleolar RNA (snoRNA)) that constitute a significant part of the transcriptome [68] as well as pseudogenes.

Pseudogenes provide a novel tier of gene regulation through the generation of endogenous silencing RNA or miRNA binding sites, which act as decoys for miRNAs [69]. Indeed some miRNAs have been demonstrated to target the genes [70]. It is hypothesised that pseudogenes act as post-transcriptional regulators of the corresponding parental gene [71]. Whilst possessing very similar sequences to their counterpart coding genes, they are unable to be transcribed due to mutation/deletion or insertion of nucleotides. Transcription of pseudogenes has tissue specificity and can be activated or reduced in disease, indicating a possible functional role in cells [72]. Interestingly, pseudogenes have been identified as increasing with age, such as pseudogene cyclin D_2 _in the ovary [73]. Whilst this study identified the differential expression of pseudogenes in cartilage of different ages, it is not known whether these are functional or have relevance to cartilage ageing. Recent work by the Encyclopaedia of DNA Elements (ENCODE) Consortium identified that 8% of the pseudogenes in the human genome are functional [74], and so with the publication of GENCODE, a reference human genome annotation for The ENCODE Project [75], more light may be shed relating to the role of pseudogenes in cartilage ageing in the near future. Pseudogenes thus present an interesting area for future research in cartilage ageing and disease.

The methodology used here does not enrich for miRNAs. To increase the identifications of small miRNAs using RNA-Seq, specific techniques are used for their enrichment in conjunction with additional miRNA abundance quantification algorithms. A single miRNA, miR-21, was however identified as increased in ageing cartilage. miRNAs are short noncoding RNAs that regulate the translation [76] and/or degradation of target message [77]. miR-21 has been implicated in inflammation [78], cancers including osteosarcomas [79], and hypomethylation [80]. The role of miR-21 in cartilage is not fully elucidated, although a study in rats found that it promoted increased proliferation and matrix synthesis in chondrocytes embedded in atelocollagen gel [81]. However, our finding is interesting because epigenetic changes such as hypomethylation occur with ageing, a risk factor contributing to several age-related pathologies [82].

A further set of small noncoding RNAs, snoRNAs - a class of small guide RNAs found in the nucleolus - were also identified in the study. The snoRNAs direct chemical modification of other RNAs, and like miRNAs are emerging as important regulators of cellular function and disease development. There are two principle classes: the C/D box snoRNAs (SNORDs) and H/ACA box snoRNAs (SNORAs), which are associated with methylation and pseudouridylation of ribosomal and other RNAs. In addition, RNase MRP and RNaseP are the only members of a further special class of snoRNAs [83]. Both were significantly reduced in older cartilage in this study. Interestingly, mutations in RNase MRP cause cartilage hair hypoplasia in which patients display dwarfism [84]. In recent work, RNase MRP was identified as a regulator of chondrocyte hypertrophy, demonstrating functional cross talk with chondrogenic pathways [85]. snoRNAs fine-tune the ribosome to accommodate changing requirements for protein production during development, normal function and disease [86]. Indeed, control of snoRNA expression may play a pivotal role in the regulation of high protein-producing cells such as chondrocytes, as demonstrated by the phenotypes of ribosomopathies [87]. Whilst there are very few studies into the significance of snoRNAS in cartilage ageing or disease, a recent study proposed the use of serum snoRNA U38 and U48 as biomarkers of early cartilage damage. These snoRNAs was detected in serum following anterior cruciate ligament injury, but were not associated with normal ageing [88]. The snoRNA transcriptome signatures in ageing cartilage provide an interesting set of genes for further studies to determine their role in ageing.

## Conclusions

A major strength of this study is that it represents the first application of RNA-Seq technology for transcriptomic studies in cartilage ageing. The study has increased our knowledge of transcriptional networks by providing a global view of the transcriptome. The molecular signatures described in this paper reflect a combination of degenerative processes and transcriptional responses to the process of ageing. This analysis further supports the use of next-generation sequencing as an ideal quantitative framework to study pathways and networks as an integrated system in order to understand the complex processes of cartilage ageing.

## Abbreviations

ADAM-TS: a disintegrin and metalloproteinase with thrombospondin; COCH: Coclin; DAVID: Database for Annotation, Visualisation, and Integrated Discovery; DKK1: Dickkopf homolog; ECM: extracellular matrix; GAPDH: glyceraldehyde-3-phosphate dehydrogenase; IL: interleukin; IPA: ingenuity pathway analysis; LINGO: leucine-rich repeat and immunoglobulin domain containing; miRNA: microRNA: MMP: matrix metalloproteinase; OA: osteoarthritis; PCR: polymerase chain reaction; RNA-Seq: RNA-sequencing; RT: real time; snoRNA: small nucleolar RNA; TGFβ: transforming growth factor beta; Wnt: Wingless and Int signalling pathway.

## Competing interests

The authors declare that they have no competing interests.

## Authors' contributions

MJP conceived the study, carried out all the laboratory work and bioinformatics analysis, and drafted the manuscript. PDC participated in the design and coordination of the study and helped to draft the manuscript. XL carried out post-processing of data, including resolution of indexes, and helped to draft the manuscript. All authors read and approved the final manuscript.

## Supplementary Material

Additional file 1 **Table S1 presenting a complete list of significantly expressed genes and DAVID analysis of the expression patterns**. The first two spreadsheets contain the DAVID results for annotation cluster analysis. The next two sheets contain the KEGG results from DAVID. Click here for file

Additional file 2 **Table S2 presenting IPA generated networks of differentially expressed genes**. Network eligible molecules were overlaid onto molecular networks, and networks were then generated based on connectivity. All identified networks and their respective molecules are tabulated. Click here for file

Additional file 3 **Table S3 presenting IPA canonical pathways**. Significant IPA canonical pathways and the associated molecules relating to these pathways. Click here for file
